# Type 1 diabetes mellitus: Roles of neutrophils in the pathogenesis

**DOI:** 10.1097/MD.0000000000036245

**Published:** 2023-12-15

**Authors:** Emmanuel Ifeanyi Obeagu, Getrude Uzoma Obeagu

**Affiliations:** a Department of Medical Laboratory Science, Kampala International University, Ishaka, Uganda; b School of Nursing Science, Kampala International University, Ishaka, Uganda.

**Keywords:** autoimmune, diabetes mellitus, neutrophils, pathogenesis, type 1 diabetes

## Abstract

Circulating neutrophil counts are reduced both in healthy autoantibody-positive individuals and in patients with type 1 diabetes, which may be related on cell-specific autoimmunity. This paper was written to give an update on roles of neutrophils in the pathogenesis of type 1 diabetes mellitus. Different research search engines like PubMed Central, Scopus, Web of Science, Researchgate, Google Scholar etc were utilised for writing this paper. A drop in blood neutrophil counts in type 1 diabetes may be caused by decreased neutrophil generation and maturation, tissue maintenance, consumption, or peripheral damage. Neutrophil count variations between studies may be explained by results from various stages of diabetes or by ethnic groups. Neutrophils can induce type 1 diabetes by colonizing pancreatic islets and interacting with other immune cells, according to exciting findings that shed new light on their role in the pathogenesis of the disease. Knowing more about the function of neutrophils in the pathogenesis of type 1 diabetes will help in early diagnosis, treatment, and even prevention of the disease.

## 1. Introduction

The first neutrophil was discovered by Paul Ehrlich in 1879.^[1,2]^ Neutrophils are produced in the bone marrow by myeloblasts. In 1893, Elie Metchnikoff named them polymorphonuclear leukocytes.^[3]^ After maturing in the bone marrow for 14 days, neutrophils can be temporarily kept in a pond. Once released into the bloodstream, they circulate as dormant cells.^[4]^ When activated, neutrophils are the first cells the body sends to the site of inflammation, serving as the first line of defence. Historically, they have been thought to as effector cells with a short lifespan (8–12 hours in the circulation and 1–2 days in tissues), low capacity for biosynthetic activity, and the ability to produce granules and reactive oxygen species (ROS).^[5,6]^ However, the advent of sensitive research methods and tools has challenged this traditional view. Recent studies have shown that neutrophils have a longer lifespan in the bloodstream (up to 5 days) than previously thought.^[5,6]^ It directs and directs the innate immune response through complex crosstalk interactions with macrophages, natural killer cells, dendritic cells, and neutrophils, most of which are important components of the inflammatory response.^[6,7]^ Neutrophils can influence gene expression in the host response to intracellular pathogens in the presence of type I interferons (IFNs).^[8]^ Once activated, neutrophils can stimulate innate immune responses by increasing phagocytosis, activating complement, and releasing soluble pattern recognition molecules that can modulate inflammation. They can also release various cytokines, neutrophil extracellular traps (NETs), and molecules that participate in innate resistance by damaging tissues and microorganisms.^[6]^ In addition to reducing inflammation, neutrophils also remove some of these cytotoxic substances that damage host cells and tissues. Neutrophils also play a role in the recruitment, programming, activation and regulation of innate and adaptive immune cells. It can also mediate the initiation of T- and B-cell-specific immunity through the use of soluble mediators or through direct intercellular communication.^[3,7,9]^

Multiple sclerosis, systemic lupus erythematosus, and antineutrophil cytoplasmic antibody-mediated vasculitis are examples of autoimmune diseases that can be complex or reactive. Numerous studies over the last few decades have shown that neutrophils play a role in the development and maintenance of autoimmune diabetes. These studies include: Monitor changes in circulating neutrophil count and function in patients with diabetes. Determine whether neutrophil activation and recruitment to pancreatic islets induces the development of autoimmune diabetes in non-obese diabetic (NOD) mice. In addition, neutrophilic infiltrates of the pancreas were found in patients with type 1 diabetes. Increased neutrophil toxicants such as neutrophil elastase, which are associated with the development of B-cell autoimmunity and diabetic complications; and the efficacy of relevant antineutrophil therapy in preventing the development of insulitis and autoimmune diabetes. Therefore, it is logical to assume that neutrophils play a role in the pathogenesis of type 1 diabetes.^[10]^

An early study found that patients with type 1 diabetes had higher neutrophil counts compared to controls, and higher neutrophil counts were associated with an increased risk of vascular disease. Studies suggest that excessive bone marrow resorption and/or neglected cell recirculation may be responsible for elevated levels of circulating neutrophils. However, recent studies have shown that the number of circulating neutrophils is reduced in both healthy autoantibody-positive patients and those with type 1 diabetes. This may be related to B-cell-specific autoimmunity. Reduced production and maturation of neutrophils, tissue sparing, peripheral consumption, or damage may contribute to reduced blood neutrophil counts in type 1 diabetes. It has been suggested that the decrease in the number of neutrophils in the blood may be explained by neutrophils located in the pancreas.^[11,12]^

There may be inconsistencies in neutrophil count changes between studies due to different stages of diabetes or ethnic groups. Further studies are needed to determine changes in neutrophil count changes, and careful and comprehensive analysis may clarify the differences. It has also been pointed out that neutrophil function changes at different stages of type 1 diabetes. Experimental data from animal models and clinical studies have clearly demonstrated consistent abnormalities in neutrophil adhesion,^[13]^ bactericidal activity,^[14–16]^ and chemotaxis.^[17,18]^ Stimuli CXC-chemokines: CXCL1, CXCL2, CXCL3, CXCL4, CXCL5, CXCL6, CXCL8, CXCL9, CXCL10, CXCL11, CXCL12. CC-chemokines: CCL2, CCL3, CCL4, CCL117, CCL18, CCL19, CCL20, CCL22. Pro-inflammatory cytokines: IL-1α, IL-1β, IL-7, IL-18, MIF. Anti-inflammatory cytokines: IL-1RA, TGF-β1, TGF-β2, Immunoregulatory cytokines: IL-10, IL-23. Colony-stimulating factors: G-CSF. Agiogenic and fibrogenic factors: HB-EGF, HGF, FGF2, TGFα, VEGF, Prokinetiein 2. Tumor necrosis factor (TNF) superfamily members: APRIL, BAFF, CD30L, RANKL, CD95L, TNF, TRAIL. Other cytokines: Amphiregulin, Midkine, Oncostatin M, PBEF. Cytokines production Neutrophils Respiratory burst NETosis Degranulation Azurophil granules (primary granules): Elastase, Proteinase-3, A1-antitrypsin, Cathepsins G, Defensisns, Lysozyme, Azurocidin, Myeloperoxidase, Bactericidal Permeability Increasing Protein. Specific (secondary granules): Lactoferrin Lysozyme, Collagenase, Gelatinase, Human cationic anti-microbial protein 18, MMP, BPIP, Neutrophil gelatinase-associated lipocalin, LL37. Gelatinase granules (tertiary granules): Gelatinase, Lysozyme. Secretory Vesicles: Alkaline phosphatase, Plasma Proteins. NETs: Extracellular deoxyribonucleic acid (DNA) with elastase, myeloperoxidase, and antimicrobial peptides. ROS: • O2–, H2O2, O2, HOCI, • OH. numerous effector molecules that are produced by neutrophils. Neutrophils have the ability to express and/or release a variety of cytotoxic substances during the maturation process and upon stimulation. During the degranulation process, 3 types of granules – azurophil granules, specific granules, and gelatinase granules – are released and are known to contain the enzyme\‘s basic building blocks. Neutrophils also have highly mobile secretory vesicles that are primarily used as a storage space for plasma membrane receptors. When neutrophils degranulate, the activity of nicotinamide adenine dinucleotide phosphate oxidase (part of cellular respiration) is activated, leading to the formation of various ROS. In addition, neutrophils can produce large amounts of cytokines, which are considered key effectors due to their broad and diverse biological activities. In addition, neutrophils can become activated and undergo NETosis (a new form of cell death) and extrude networks of extracellular fibrils called neutrophil extracellular traps (NETs). APRIL, a proliferation-inducing ligand; BAFF, B cell activating factor; BPIP, bactericidal permeability-increasing protein; DNA, deoxyribonucleic acid; EGF, epidermal growth factor; G-CSF, granulocyte colony-stimulating factor; HB-EGF, heparin-binding epidermal growth factor; HGF, hepatocyte growth factor; IL-1RA, interleukin-1 receptor antagonist; MIF, macrophage migration inhibitory factor; PBEF, pre-B cell colony growth factor; RANKL, receptor activator of nuclear factor-ligand jB; TGF, transforming growth factor; VEGF, vascular endothelial growth factor; TRAIL, tumor necrosis factor-related apoptosis-inducing factor. However, there is currently conflicting information in the literature regarding other host defense functions such as neutrophil oxidative burst activity,^[16,19,20]^ migration,^[14,17,21]^ phagocytosis^[14–16,22]^ and apoptosis.^[21,23,24]^ Altered neutrophil function may result from altered receptors,^[25]^ altered calcium signaling,^[26]^ and altered neutrophil adenosine triphosphate synthase activity.^[27]^ HbA1c levels and infection history did not affect neutrophil function in patients with type 1 diabetes, but the rate of neutrophil apoptosis was significantly correlated with HbA1c levels in patients with type 2 diabetes.^[22]^ According to the report, people with short-term diagnosed type 1 diabetes and pre-diabetes may have a significant decrease in neutrophil counts, but people with long-term diagnosed type 1 diabetes do not. This suggests that neutrophil function may be closely related to B cell autoimmunity. Although the impact of environmental factors such as hyperglycemia and advanced glycation end products (AGEs) on neutrophil function is well known, little is known about the relationship between genes and dysfunction neutrophils. Although highly correlated with the pathogenesis of type 1 diabetes, it has been hypothesized that genes related to human leukocyte antigen D are not associated with reduced neutrophil function.

## 2. Neutrophil-derived effector molecules and type 1 diabetes

Adhesion molecules Adhesion molecules, which occur in both soluble and membrane-bound forms, are essential for the migration of neutrophils from the bloodstream to target tissues^[28]^ and play a role in immune surveillance.^[29]^ In autoimmune diabetes, immune cells must travel from the blood vessels to the pancreas to function properly.^[30]^ Many studies have revealed the role of 2 families of adhesion molecules, including integrins and selectins, in the pathogenesis of type 1 diabetes. P-selectin (CD62P, GMP-140), L-selectin (CD62L), and E-selectin are family members selectin.^[31]^ L-selectin is mainly expressed on the surface of various immune cells. Immediately after activation, neutrophils are rapidly eliminated.^[32]^ Changes in the levels of soluble L-selectin have been observed in patients with type 1 diabetes and in the preclinical stages of the disease.^[33]^Early studies suggested that L-selectin may be one of the emerging risk factors for the development of type 1 diabetes in humans and animal models.^[34]^ A follow-up study showed increased levels of sL-selectin and high antibody titers for insulin-related protein 2 in the liver of children with type 1 diabetes^[35]^ as well as in children with diabetes.^[28]^ Consequently, increased sL-selectin in patients may indicate an active process of destructive insulitis. Increased levels of sL-selectin are also associated with seroconversion to autoantibody positivity, suggesting that leukocyte activation and b-cell autoimmunity are linked events. However, the early onset of B-cell autoimmunity cannot be fully explained by the high concentration of circulating adhesion molecules when viewed from a prospective perspective (10 and 2 years, respectively).^[36,37]^ No differences were found in the integrated concentration of sL-selectin associated with different specificities and titers of autoantibodies. Integrins, single chains and heterodimers with a common b chain consist of integrin b and integrin b2. The three b2 integrin complexes include CD11a (leukocyte antigen-1 [LFA]), CD11b (macrophage-1 [MAC-1]), and CD11c (p150.95). LFA is expressed on neutrophil membranes and the MAC-1 glycoprotein complex is retained in secondary granules.^[4]^ LFA-1 has been shown to be involved in the initiation of insulitis in humans and animal models of type 1 diabetes.^[38,39]^ Treatment with monoclonal antibodies against LFA-1 can delay the spontaneous onset of the disease in NOD mice.^[40–43]^

### 2.1. Neutrophil counts and functions in autoimmune type 1 diabetes

Inhibiting the ability of effector cells to migrate to the pancreas appears to be a preventive mechanism. It is known that LFA-1 can be expressed in the membranes of lymphocytes, monocytes and natural killer cells in addition to neutrophils. To our knowledge, no study has investigated the expression of LFA-1 on neutrophils in type 1 diabetes. Several studies have shown that LFA-1 is expressed in monocytes.^[44]^ In a pilot study, type 1 diabetes patients with newly diagnosed disease had low levels of LFA-1 alpha chain expression and normal levels of LFA-1 b chain positive monocytes.^[45]^ In another study, LFA-1 expression on monocytes was higher in patients with overt diabetes compared to controls, but there was a positive correlation between LFA-1 expression and islet cell autoantibody (ICA) titers. This suggests that LFA-1 plays an important role in the pathogenesis of type 1 diabetes. MAC-1, another b2 integrin, can promote thrombosis and mediate leukocyte vascular infiltration. It is therefore believed to be related to the frequency of vascular events.^[46,47]^ Patients with type 1 diabetes have higher levels of MAC-1 receptor expression on neutrophils, regardless of diabetes severity or HbA1c level. Acute hyperglycemia is a factor that increases MAC-1 expression, and neutrophil actin polymerization may also play a role. It has been shown that increased expression of MAC-1 may contribute to the development of diabetes complications by increasing the adhesion between neutrophils and endothelial cells.^[48,49]^ Degranulation products Neutrophils are complex cells that loosen and release a variety of granules and vesicles, including neutrophil granules, specific granules, gelatinase granules, and secretory vesicles. Typically, various proteases produced during neutrophil maturation are stored as intracellular granules and are released into the extracellular space after neutrophil activation and degranulation. Neutrophil proteases can be active in both membrane-bound and soluble forms, the latter acting extracellularly in tissues and plasma.^[50]^ Neutrophil elastase and myeloperoxidase, found in azurophil granules, are 2 proteases thought to be relatively specific to neutrophils. Although it can be secreted by a variety of cells, plasma lactoferrin, a component of specialized granules, is mainly derived from neutrophils and is thought to be closely related to the number of circulating neutrophils. Most extracellular matrix and plasma proteins can be degraded by neutrophil elastase.^[51]^ In addition, neutrophil elastase can regulate inflammation by lysing various substances, including cytokines, chemokines, and cell surface receptors. Plasma and total neutrophil elastase levels were found to be significantly higher in patients with type 1 diabetes compared to controls. Because of its potential contribution to the development of vascular disease, elevated plasma neutrophil elastase concentrations may also be used as a predictor of complications such as diabetic angiopathy and coronary artery disease.^[52]^ In addition, previous studies in our laboratory have shown a positive correlation between circulating levels of neutrophil elastase produced by activated neutrophils and the number and titer of autoantibodies against B-cell-specific antigens, suggesting neutrophil activation and suggesting increased protease activity. It may happen. role. The role of the antibiotic in the development of B-cell autoimmunity. Therefore, serum neutrophil elastase may be a reliable biomarker for the early detection and prediction of type 1 diabetes.^[53]^ While some studies have suggested an association between elevated plasma neutrophil elastase levels and poor long-term glycemic and metabolic control short in patients with type 2 diabetes,^[54–59]^ other studies have suggested an association between elevated plasma neutrophil elastase levels and poor short-term glycemic and metabolic control. There appears to be no correlation. Type 1 diabetes. Patient and age, white blood cell count, HbA_1_c, plasma glucose or duration of diabetes.^[60,61]^ A powerful oxidant called myeloperoxidase (MPO), which constitutes about 5% of total neutrophil proteins, is found mainly in primary granules. MPO is important for understanding oxidative stress because it catalyzes the production of ROS, which can promote atherosclerosis and modify lipids and proteins.^[62,63]^ Diabetic patients can inhibit MPO activity, reducing the efficiency of neutrophils to phagocytose bacteria and making them more susceptible to infection.^[64]^ found no significant differences in plasma MPO levels in children and adolescents with type 1 diabetes compared to controls. However, another study found that serum MPO concentrations in diabetic children were significantly higher than those in healthy controls. This finding may mean that people with type 1 diabetes are at increased risk of cardiovascular disease. There was no significant correlation between duration of diabetes, HbA1c level and actual blood glucose level regardless of increased or decreased MPO level.^[65]^ Cytokines - In addition to synthesizing a wide range of polypeptides directly involved in effector functions, neutrophils can produce numerous proteins that regulate inflammation either constitutively or synchronously.^[66,67]^ Cytokines are considered the most important effectors among these molecules due to their broad and diverse biological activities.^[66,68]^ When neutrophils are activated, cytokines are temporarily stored in intracellular pools and then rapidly released.^[69]^ Human and mouse neutrophils are known to differ in their ability to express cytokines, particularly interleukin (IL)-6, IL-17A, IL-17F, and IFN-c6. The state of the cell and the type of stimulus determine whether activated neutrophils release cytokines. Although it is clear that neutrophils produce much smaller amounts of cytokines than other immune cells, it is important to remember that neutrophils represent the majority of cells infiltrating inflamed tissues and can be considered an important source of cytokines.^[69]^

Given the wide range of biological activities exhibited by cytokines, it is logical to assume that neutrophils play a central role in both innate and adaptive immune responses. Neutrophils also play an important role in inflammatory responses.^[70]^ Many cell types produce local cytokines during insulitis, and these cytokines have been implicated in the pathogenesis of autoimmune diabetes. It has been suggested that cytokines such as TNF-α, IL-1, IFN-c, and TNF-b can stimulate the production of toxic free radicals that can initiate the destruction of pancreatic b cells. In addition, IL-1 produced by macrophages can impair b-cell function and reduce b-cell mass. In addition, cytokines such as IFN-c, IL-1, and TNF-α can worsen autoimmunity by enhancing the adaptive immune response. In destructive B-cell insulitis, the expression of the cytokines IFN-α, TNF-α and IL-1 is increased.^[71–73]^ Elevated levels of IL-18 are associated with the presence of multiple autoantibodies in patients with recent-onset type 1 diabetes. Because of their important role in disease, serum cytokines are promising candidates for additional markers and monitoring interventions beyond autoantibodies to predict type 1 diabetes.^[74]^ However, there are many problems and contradictions. The main problem is that most cytokines do not have normal ranges. It is also important to consider physiological differences as well as differences in technical testing methodology and testing bias. Some cytokines have normal baseline values and their levels can be measured in plasma using current technologies, but lack of disease specificity is another obstacle.^[75]^ Finally, cytokine concentrations change, especially in the presence of infections, allergies, and drugs. Although neutrophils can produce a variety of cytokines, and cytokines have a wide range of biological functions, it is unclear how cytokines produced by neutrophils contribute to the development and progression of autoimmune diabetes. ROS induces the production of ROS, such as nicotinamide adenine dinucleotide phosphate (NADPH) oxidase activity, superoxide anion, hydroxyl radical, and hydrogen peroxide, which are components of cellular respiration. The onset of NADPH oxidase activity in neutrophils occurs almost simultaneously with degranulation and is delayed by approximately 20 seconds. Neutrophils are a rich source of toxic oxygen species.^[76]^ ROS production from neutrophils has been reported to be increased in type 1 diabetes patients and mouse models. High levels of these harmful substances not only cause the death of pancreatic B cells, but also weaken the ability of neutrophils to fight free radicals, putting diabetics at risk of infections. One theory suggests that lipid peroxidation, platelet and neutrophil activation, glycation, and glycation are responsible for increased ROS production in type 1 diabetes.^[77]^ Although ROS alone cannot improve respiration, it appears that they can stimulate neutrophil ROS production by upregulating of NADPH oxidase and neutrophil stimulation. However, other researchers found reduced respiratory responses and showed that diabetic patients had reduced neutrophil NADPH oxidase activity and reduced superoxide production in vitro. High glucose levels rapidly reduce ROS produced by activated neutrophils by inhibiting glucose-6-phosphate dehydrogenase. The results of different ROS levels in these studies may be due to the different metabolic status and disease duration of the patients.^[78–81]^ Many studies have shown that, in addition to neutrophils, ROS are also produced by macrophages, mesangial cells, and glomerular epithelial cells. Infiltration of islet-derived ROS produced by macrophages and damage to islet b cells have been shown to induce autoimmune diabetes in NOD mice. However, it is currently unknown what function ROS produced by neutrophils play in the pathology of type 1 diabetes. The formation of fibrillar extracellular networks (NETs) by neutrophils in response to various stimuli is called NETosis, a new form of cell death that is basically different from apoptosis and necrosis. In addition to the production of classical effector molecules such as proteases, cytokines and ROS, extracellular traps for neutrophils are also formed by neutrophils by NETs. NETs, which are useful in antibacterial processes, are composed of nuclear components decorated with granule proteins and short peptidoglycan recognition proteins.^[82–87]^ In addition, NETs are associated with autoimmunity because they produce extracellular automolecules.^[3]^ Type 1 diabetes, small vessel vasculitis, and systemic lupus erythematosus are just a few of the autoimmune diseases associated with dysregulated NET formation and NETosis.^[88–90]^ Many studies have shown that increased NETosis in patients with systemic lupus erythematosus can trigger autoimmunity and cause vascular disease.^[91]^ However, the volume of research on NETs in the pathogenesis of autoimmune diabetes is very limited. NET formation in pancreatic islets was observed 2 weeks after birth in young NOD mice.^[92]^ Increased NET formation was found to be closely associated with increased levels of circulating neutrophil elastase in patients with autoimmune type 1 diabetes, suggesting that this may be important in initiating b-cell autoimmunity. However, conflicting data suggest that neutrophils from patient’s diabetics produce lower amounts of NETs than neutrophils from healthy individuals. This is because high glucose conditions can inhibit and delay neutrophil NET formation. As a result, NETs expressed fewer neutrophils with antibacterial properties, which partially explained the increased susceptibility to infections in diabetic patients.^[93,94]^

## 3. Cell–cell interactions in type 1 diabetes

Neutrophils and other immune cells in addition to changing their phenotype in response to signals from the local environment, neutrophils participate in complex bidirectional interactions with other immune cell types.^[95]^ Depending on the context, these interactions can directly or indirectly affect the activation, maturation and effector functions of other immune cells. They provide instructions to other immune cells by releasing cytokines, granules and ROS.^[96]^ You can also participate in mobile communication networks. Most of the immune cells responsible for the inflammatory response and its escalation are neutrophils, which interact with other cells. Interactions between pancreatic B cells and immune cells, such as neutrophils and other immune cells, play an important role in the development of type 1 autoimmune disease.^[97]^ Young NOD mice are able to recruit and circulate activated neutrophils, B-1a cells, and plasmacytoid dendritic cells as a result of physiological B-cell death. This crosstalk between innate immune cells was found to occur in the pancreas and is essential for the development of type 1 diabetes, and another novel crosstalk between macrophages and B cells was found to be the cause in the pancreas. Pancreatic infiltration with neutrophils in the early stages of autoimmune diabetes. Large-scale studies have shown that T cells are strongly involved in the pathogenesis of type 1 diabetes in both animal models and humans.^[98]^The most common type of immune cell that infiltrates the pancreas during insulitis is CD8 T lymphocytes in the early stages of diabetes.^[99]^ They can stimulate adaptive immune responses and damage pancreatic b cells through pathogenic cytotoxicity regulated by major histocompatibility complex class I molecules, and both CD4 and CD8 T cells directly or indirectly induce b cell apoptosis.^[100]^ They can release cytokines that can promote CXCR2 ligands in NOD mice play an important role in regulating neutrophil migration to pancreatic islets, and production of the chemokines CXCL1 and CXCL2 by macrophages and b cells attracts neutrophils. Blocking CXCR2 can lead to a rapid decline in islet-specific CD8 T cells at early stages, which may not only suppress subsequent diabetes-induced T cell responses, but also suppress the subsequent development of diabetes.^[101,102]^ Recently, decreased expression of Cxcr1 messenger ribonucleic acid was found in both neutrophils and CD4 T lymphocytes isolated from NOD mice compared to diabetes-resistant mice. Low levels of Cxcr1 expression in NOD mice may play an important role in the development of autoimmune diabetes. In addition, patients with type 1 diabetes may have circulating IL-17b-specific autoreactive CD4 T cells at diagnosis. As a result of this IL-17 proinflammatory state, neutrophils can be recruited and infiltrate the pancreatic tissue. Both neutrophils and T cells appear to play diverse, synergistic roles in the pathogenesis of type 1 diabetes. Therefore, it is important to pay attention to the interaction between neutrophils and T cells in the development of type 1 diabetes. Neutrophils attach to endothelial cells and readily enter pancreatic islets, damaging them and promoting infiltration. However, most studies of neutrophil adhesion to endothelial cells in diabetes focus on the development of atherosclerosis-mediated diseases such as diabetic microvascular and macrovascular complications,^[103,104]^ and the study of neutrophil adhesion to type 1 endothelial cells has been adjusted. The pathogenesis of diabetes is relatively small. Several endothelial adhesion molecules, such as intercellular adhesion molecule-1 (ICAM-1), CD62E, CD62P, and vascular adhesion molecule-1, can attract circulating neutrophils and then bind to neutrophil adhesion. ICAM-1, the best characterized cell surface adhesion molecule, has been shown to be involved in the pathogenesis of type 1 diabetes by participating in the extravasation of circulating leukocytes into the inflamed pancreas. ICAM-1 expression has been reported to be increased in vascular endothelial cells of pancreatic islets of NOD mice as well as in humans with new-onset type 1 diabetes. Compared with healthy controls, serum levels of circulating ICAM-1 were also significantly increased in patients with recent-onset type 1 diabetes and their first-degree relatives.^[105,106]^ Among these subjects, soluble ICAM-1 levels in prediabetic patients with positive autoantibodies were relatively higher than those in clinical diabetic patients and were positively correlated with levels of ICA and glutamic acid carboxylase autoantibodies.^[58]^ This, together with the study on neutrophil infiltration of pancreatic islets mentioned above, suggests that the interaction between neutrophils and endothelial cells is definitely involved in the accumulation of inflammatory neutrophils in pancreatic islets and their destruction. It is not clear what controls the process. Neutrophils, platelets, and leukocytes (the latter composed of neutrophils) have been shown to regulate and influence each other through platelet–leukocyte interactions and the release of soluble effector mediators.^[107–112]^ Activated platelets express selectins, inflammatory cytokines, and chemokines, promoting neutrophil activation and recruitment. Conversely, apoptosis and activated leukocytes can promote platelet aggregation and adhesion. Adhesion of activated platelets to leukocytes is involved in the development of thrombotic events, and apoptotic leukocytes can induce thrombotic currents.^[113]^ found increased aggregation and cross-linking of platelets and white blood cells in the blood of patients with type 1 diabetes. Promotion of leukocyte-platelet interactions in the disease may be associated with increased levels of plasma elastase, which may induce platelet activation and increases production of important soluble mediators such as platelet-activating factor and superoxide anion.^[114–117]^ AGE-BSA, a model compound for AGEs, can induce neutrophil apoptosis and increase platelet-neutrophil aggregation by upregulating Mac-1 expression. Because leukocyte–platelet interactions link inflammation to thrombosis and may contribute to vascular occlusion and tissue ischemia,^[118,119]^ increased platelet–leukocyte aggregation and interactions in patients with type 1 diabetes may contribute to platelet hyperactivity. You can contribute. Occurrence of microvascular complications.^[117]^ It has also been shown that increased aggregation of platelets and neutrophils in diabetic patients may be one of the factors causing serious cardiovascular diseases.^[120]^

## 4. Antineutrophil therapy

Previous studies have shown that neutrophils play an important role in the progression of type 1 autoimmune diseases, raising the possibility that neutrophils may be a target for intervention. Neutrophil activation, endothelial binding, transendothelial migration, islet migration, and release of cytotoxic products are potential targets for pharmacotherapy. You can. Anti-neutrophil treatments in diabetes include direct inhibition of neutrophil recruitment and anti-adhesion therapies. Recruitment of neutrophils from the circulation to the pancreas is required before these cells can enter the pancreatic islets and exert their effects. Macrophages and b cells have been reported to attract CXCR2-expressing neutrophils by producing the chemokines CXCL1 and CXCL2. Inhibition of neutrophil recruitment by CXCR2 antagonists can reduce diabetic T cell responses and prevent further development of autoimmune diabetes. As mentioned above, the attachment of neutrophils to the endothelial veins of the pancreatic compartment is a critical first step in the inflammatory response and is considered one of the most important steps in the initiation of the inflammatory response. Onset of autoimmune diabetes. This prevents differential adhesion. Beta cells B-1a cells p-DC cells Neutrophils Endothelial cell transformation and activation Recruitment of pancreatic infiltrates Vascular complications related to diabetes Initiation of type 1 diabetes Initiation of diabetic T cell response and recruitment of B-1a cells Macroplatelets T cells IFN-α production Activation and stimulus recruitment Stimuli activate neutrophils to release SRMP.^[98]^

### 4.1. The mechanism of the initiation and pathogenesis of type 1 diabetes

It is not yet known how type 1 diabetes develops and how it progresses. The disease was thought to be caused primarily by the physiological death of immune cells, especially B cells, which can recruit and activate neutrophils to infiltrate the pancreatic islets. In the pancreas, neutrophils have the ability to produce CRAMP, which is activated by immunoglobulin G, which is specific for DNA secreted by B-1a cells. Plasmacytoid dendritic cells can be stimulated by immunoglobulin G and the CRAMP peptide complex to produce interferon-α as well as B cell debris such as spontaneous deoxyribonucleic acid. The aforementioned crosstalk between these immune cells is required to activate the diabetogenic T-cell response, which ultimately leads to the development of type 1 diabetes. In addition, interactions between neutrophils and other non-immune cells, such as platelets in blood or vascular endothelial cells, play an important role in diabetic microvascular and macrovascular complications. Conversely, metabolic changes in patients with type 1 diabetes may activate and damage neutrophils. Advanced glycation end products (AGEs) and interferon (IFN) are 2 terms. Molecules such as integrins and selectins expressed by specific neutrophil antagonists may be an effective way to inhibit the development of insulitis by reducing neutrophil migration to the inflamed pancreas. Anti-L-selectin and anti-VLA-4 antibodies have been shown to delay the development of insulitis by preventing leukocyte adhesion to inflamed blood vessels and revealing leukocyte recruitment to islets.^[41]^ showed that administration of anti-α4 integrin and/or anti-LFA-1 antibodies inhibited b-cell destruction and protected NOD mice from spontaneous and adoptive diabetes.^[40,43,121,122]^ Combination therapy using 2 monoclonal antibodies can prolong the onset of diabetes46. Furthermore, long-term inhibition of LFA-1 and α4 integrins not only prevents diabetes during treatment, but also induces persistent resistance to the condition long after the intervention has ceased. Demonstrates effector cell function. It activates suppressor cells involved in immune regulation. 46 In addition, short-term blockade of the LFA-1/ICAM-1 pathway at a critical stage was shown to induce specific peripheral tolerance to b-cell Ag(s) in NOD mice at an early age, providing complete protection. Onset of autoimmune diabetes. Therefore, type 1 diabetes is mainly caused by adhesion molecules such as L-selectin, VLA-4 and LFA-1, and the disease can be stopped by interfering with these adhesion pathways. However, adhesion molecules are known to be expressed not only in neutrophils but also in lymphocytes, monocytes and natural killer cells. Studies have shown that anti-LFA-1 therapy can mitigate neutrophil-related damage by reducing neutrophil adhesion, aggregation, aggregation, and tissue infiltration. In addition, activation of the neutrophil respiratory burst can be limited by anti-LFA-1 inhibition.^[123–129]^ Therefore, careful research is needed to better understand how neutrophil-targeted anti-adhesion therapy can prevent the progression of spontaneous diabetes.

## 5. Conclusion

Research over the past few decades has shown that neutrophils are critical for the onset and progression of autoimmune type 1 diabetes. Exciting new findings have illuminated the roles played by neutrophils in the development of type 1 diabetes. Specifically, it has been shown that neutrophils may induce the disease by infiltrating the pancreatic islets and interacting with other immune cells. Remarkably little research has been done explicitly on the connection between neutrophils and diabetes, despite the fact that neutrophils are known to be the most common cell type in the circulation and the first cells drawn to the site of inflammation. Improved understanding of neutrophils’ role in the pathophysiology of type 1 diabetes will aid in early disease detection, therapy, and potentially prevention.

## Author contributions

**Conceptualization:** Emmanuel Ifeanyi Obeagu.

**Methodology:** Emmanuel Ifeanyi Obeagu, Getrude Uzoma Obeagu.

**Supervision:** Emmanuel Ifeanyi Obeagu.

**Validation:** Emmanuel Ifeanyi Obeagu, Getrude Uzoma Obeagu.

**Writing – original draft:** Emmanuel Ifeanyi Obeagu, Getrude Uzoma Obeagu.

**Writing – review & editing:** Emmanuel Ifeanyi Obeagu, Getrude Uzoma Obeagu.
